# A Logical Model of Homology for Comparative Biology

**DOI:** 10.1093/sysbio/syz067

**Published:** 2019-10-09

**Authors:** Paula M Mabee, James P Balhoff, Wasila M Dahdul, Hilmar Lapp, Christopher J Mungall, Todd J Vision

**Affiliations:** 1 Department of Biology, University of South Dakota, 414 East Clark Street, Vermillion, SD 57069, USA; 2 Renaissance Computing Institute, University of North Carolina, 100 Europa Drive, Suite 540, Chapel Hill, NC 27517, USA; 3 Center for Genomic and Computational Biology, Duke University, 101 Science Drive, Durham, NC 27708, USA; 4 Division of Environmental Genomics and Systems Biology, Lawrence Berkeley National Laboratory, Berkeley, CA 94720, USA; 5 Department of Biology and School of Information and Library Sciences, University of North Carolina at Chapel Hill, Chapel Hill, NC 27599-3280, USA

## Abstract

There is a growing body of research on the evolution of anatomy in a wide variety of organisms. Discoveries in this field could be greatly accelerated by computational methods and resources that enable these findings to be compared across different studies and different organisms and linked with the genes responsible for anatomical modifications. Homology is a key concept in comparative anatomy; two important types are historical homology (the similarity of organisms due to common ancestry) and serial homology (the similarity of repeated structures within an organism). We explored how to most effectively represent historical and serial homology across anatomical structures to facilitate computational reasoning. We assembled a collection of homology assertions from the literature with a set of taxon phenotypes for the skeletal elements of vertebrate fins and limbs from the Phenoscape Knowledgebase. Using seven competency questions, we evaluated the reasoning ramifications of two logical models: the Reciprocal Existential Axioms (REA) homology model and the Ancestral Value Axioms (AVA) homology model. The AVA model returned all user-expected results in addition to the search term and any of its subclasses. The AVA model also returns any superclass of the query term in which a homology relationship has been asserted. The REA model returned the user-expected results for five out of seven queries. We identify some challenges of implementing complete homology queries due to limitations of OWL reasoning. This work lays the foundation for homology reasoning to be incorporated into other ontology-based tools, such as those that enable synthetic supermatrix construction and candidate gene discovery. [Homology; ontology; anatomy; morphology; evolution; knowledgebase; phenoscape.]

Distinguishing homology, that is, similarity due to inheritance from a common ancestor, from similarities that arise independently, is the foundation of the comparative approach that is applied across many different fields of biology. Comparative genomics, for instance, has led to the identification of homologous patterns of gene activity and regulation that have been conserved over hundreds of millions of years of evolution. This has been aided considerably by computer-based analysis, which is enabled by the standardization of genomic data. The longer tradition of comparative anatomy has also revealed extensive conservation, with the homologies between the jaw bones of fishes and the inner ear bones of mammals as a quintessential example. The complexity of anatomical data, however, has been an impediment to standardization and computation, and many of the critical tasks rely on manual inspection of the data and human judgment (Vogt 2018a). Advances in this area have been made using semantic reasoning but these have not explicitly incorporated nor evaluated homology reasoning. Here, we formalize the biological expectations for homology reasoning and evaluate the consequences of applying formal homology relationships between anatomical structures in an anatomy ontology, using the skeletal elements of the fins and limbs of vertebrates as an example.

Anatomy ontologies are formal graph representations of anatomical structures and the relationships among them. They provide the foundation for computational analyses of comparative anatomy data that are semantically aware. By aggregating expert knowledge of different anatomical structures and organisms, they are a key resource for comparative analysis. Anatomy ontologies exist today that can connect the anatomical features and linked data from millions of biological species.

Our motivation for undertaking this work, and the context in which we test different formalisms, is the Phenoscape project (phenoscape.org), in which we have been working to demonstrate the value of a semantic approach through the development of multispecies anatomy ontologies (Dahdul et al. 2010, 2012; Mungall et al. 2012; Haendel et al. 2014) and other required resources including taxonomy ontologies (Midford et al. 2013), annotation tools (Balhoff et al. 2010, 2014), and a knowledgebase to hold these structured data (Mabee et al. 2018). Phenoscape has annotated $>$22,000 anatomical character states to $>$5000 vertebrates from the comparative evolutionary literature and integrated the resulting more than half a million taxon phenotypes with approximately 400K gene phenotype annotations from model organisms (zebrafish, mouse, *Xenopus*, and human) into its knowledgebase. Using ontology-based reasoning to integrate taxon and gene phenotypes, the team has demonstrated the discovery of candidate genes underlying evolutionarily novel phenotypes (Edmunds et al. 2016) and the use of semantic similarity to discover evolutionary variation related to gene phenotypes (Manda et al. 2016).

The anatomy ontologies and reasoning capabilities of the Phenoscape Knowledgebase (hereafter, the KB) provide the core framework for automatically extracting the basic data desired at the outset of a comparative anatomical study, namely all of the published data for a set of anatomical structures in a focal taxon. Although a researcher might use these data in a number of different ways, the data required will generally be a matrix of taxa and anatomical phenotypes. An illustration of such reasoning is provided by the OntoTrace tool, which can directly extract or infer the presence or absence of anatomical entities across all studies in the KB for a user-specified set of anatomical entities and taxa; these can then be aggregated into an aligned matrix for downstream analysis (Dececchi et al. 2015; Jackson et al. 2018).

To date, reasoning performed in the KB using anatomy ontologies has not explicitly incorporated homology. The present work attempts to address that deficiency, but it is not a straightforward task, in part because homology can be defined in numerous ways. The literature is replete with continuing discussions about types of homology, levels of homology, and how to distinguish homology from homoplasy, that is, similarity that is not due to common ancestry (convergent or parallel evolution; Bock 1969; Roth 1984, 1988; Wagner 1989; Hall 2003, 2012; Cracraft 2005; Scotland 2010; Minelli and Fusco 2013; Roux et al. 2015; Briscoe and Ragsdale 2018; Kratochwil et al. 2018; Ochoterena et al. 2019). The similarity of features that are descended from a common ancestor is typically referred to as “phylogenetic homology” or “historical homology” (Wagner 1989); the homologies between the jaw bones of fishes and the inner ear bones of mammals are a quintessential example (Romer 1950). “Serial homology,” a type of iterative homology, is the historical and developmental relationship among segmented or, more generally, iterated, structures within an organism, for example, across the various appendages of crustacea, the vertebrae of vertebrates, and the arms of a starfish (Roth 1984). Despite the volume of literature on homology, an explicit mapping from the biological understanding of these types of homology relationships to their downstream logical consequences has not been made. For instance, given an assertion of serial or historical homology between two anatomical structures, how should that knowledge be logically propagated to their parts, subtypes, or developmental precursors? Having explicit logic that mirrors biologists’ expectations but that can also be employed computationally would enable computationally assisted discoveries in comparative biology that are limited only by the scale of available semantically described biodiversity data.

## How to Accommodate Homology within Ontologies?

A number of approaches to using information about homology in relating anatomical entities or phenotypes in ontologies have been proposed or implemented. Early ideas for incorporating homology grew out of the effort to expand the taxonomic scope of anatomy ontologies beyond the single species, typically model organisms, for which many of them had been designed (Mabee et al. 2007b). It was argued that because similarity of phenotype frequently owes to the continuity of inherited information, that is, homology, that it must be accommodated in any attempt to create multispecies anatomy ontologies (Mabee et al. 2007b). Several approaches were considered, one of which was to represent homologs with different names as synonyms of a single anatomical entity (Mabee et al. 2007a). For example, the series of bones located along the midline between the skull and dorsal fin in different fish species is referred to by different names (“supraneural” and “predorsal”). Given the homology of these series across all fishes (Mabee 1988), they are represented in the Uberon anatomy ontology by a single concept under the term “supraneural” with the exact synonym “predorsal.” Although this representation might suffice for some structures, it does not accommodate differently named structures with very different structural, developmental, and positional relationships. For example, the stapes, an inner ear bone in mammals, is the undisputed homolog of the hyomandibula, a jaw bone in fishes. If these terms were synonymized, many specific relationships would need to be generalized for the ontology to accurately represent both the stapes and hyomandibula. Synonymizing also does not suffice in cases where the homology is uncertain. For example, the “alular digit” (the first digit in bird wings) is considered by many, on the basis of paleontological evidence (Wagner and Gauthier 1999), to be a homolog of the first digit in other vertebrates (the “manual digit 1”). However, these are typically not considered homologous on the basis of developmental data (Burke and Feduccia 1997; Feduccia 1999).

Another proposed approach was to represent hypotheses for the homology of anatomical entities outside of formalized ontologies; the ontology itself could remain homology-neutral (Mabee et al. 2007a; also see Vogt 2008, 2018a; Vogt et al. 2009; Dahdul et al. 2010, 2012). Anatomical entities would be defined on the basis of spatio-structural properties that would allow their unambiguous identification and re-identification exclusively on the basis of anatomy (Vogt 2018a). This approach is further justified by the fact that at least some homology hypotheses are too weak or controversial to be embedded in the ontology in the same way as the hardened knowledge concerning the types and parts of anatomical structures. This approach, to capture hypotheses of homology independently of structural and functional information, was supported by Haendel et al. (2008), who further proposed a new relationship, *homologous_to*, to be included in the OBO Relations Ontology. This relationship was defined and formalized, along with *not_homologous_to* by Travillian et al. (2011), who implemented them in the Vertebrate Bridging Ontology (VBO). The VBO was introduced to enable the transfer of information about homologous anatomical structures between species-specific anatomical ontologies, and a beta version was integrated into the Experimental Factor Ontology (Malone et al. 2010) to support cross-species comparisons of orthologous genes in homologous tissues through the Gene Expression Atlas interface.

The Bgee initiative (bgee.org) led computational work to use homology relations to align anatomical entities between species-specific anatomy ontologies to enable comparisons of gene expression patterns between species (Bastian et al. 2008; Parmentier et al. 2010; Roux et al. 2015). These authors designed an algorithm, implemented in the software Homolonto (Parmentier et al. 2010), to create new relationships between anatomical ontologies, and a homology ontology (HOM; Roux and Robinson-Rechavi 2010) to clarify homology-related concepts. They later developed the vertebrate Homologous Organs Groups ontology (vHOG), a multispecies anatomical ontology for vertebrates based on the homologous organ systems used in the Bgee database of gene expression evolution (Niknejad et al. 2012). vHOG describes structures with historical homology relations between model vertebrate species. It includes manually reviewed mappings to species-specific anatomical ontologies; no homology hypotheses are stated within the ontology itself (Niknejad et al. 2012).

Currently, in multispecies anatomy ontologies such as Uberon (Haendel et al. 2014), TAO (Dahdul et al. 2010), and VSAO (Dahdul et al. 2012), the definitions of classes focus on some re-identifiable property or properties that members of the classes have in common; they do not include criteria of homology. Most often these properties involve structural criteria but developmental and functional ones are employed, too. A class such as “endochondral bone” (UBERON:0002513) reflects the developmental similarity of its subtypes, “long bone” (UBERON:0002495) reflects structural similarity, and “eye” (UBERON:0000970) reflects functional similarity. This pluralistic approach reflects the multiple ways that comparative morphologists understand and group anatomical structures. By not imposing homology on the ontology, one might argue that broader possibilities for data discovery are enabled. Simply, searches for similar anatomical structures are not constrained by the special similarity owing to shared evolutionary descent.

That notwithstanding, for many classes in multispecies anatomy ontologies, and therefore Uberon (Haendel et al. 2014), homology is implicit in their semantics, as evidenced by how they are applied in practice. For example, the most proximal bone of the forelimb/arm in vertebrates, including humans, is named the “humerus.” The humerus is considered a historically homologous bone across vertebrates, and the single label for this bone in Uberon, “humerus,” signifies homology in this case. Expert curators use this term across vertebrates without restricting the semantics to different taxonomic groups. Homology, in fact, is similarly woven into the names of many if not most anatomical structures (Bastian et al. 2008), and a multispecies anatomy ontology, therefore, cannot be characterized as “homology-free” (Manda et al. 2016). That said, the definitions of these ontology terms do not explicitly reference evidence for homology, for example, particular phylogenetic hypotheses and/or morphological data, and thus in that sense homology is not a formal part of the semantic framework. In fact, the assumption that commonly named structures such as “humerus” are homologous because they share a common term definition that may reference a type of shared similarity is insufficient, as homology hypotheses ultimately require testing with phylogeny.

Finally, some arguments have been made to bake homology into the ontology (Franz and Goldstein 2013), embodying the knowledge derived about character state homology from phylogenetic trees. Although this is a solution for well-supported hypotheses of homology, for others that are disputed, it is not. Further, because proposals of homology are tested by concordance with phylogeny, to the extent that phylogenetic hypotheses themselves are in flux, hypotheses of homology are as well.

Our goal with this study is to understand the ramifications of different ways of representing historical and serial homology for anatomical entities as a set of ontology axioms. We first describe two alternative models which differ in their requirements for logical expressivity. We then assess the implications of these differing models when applied to the classic example of homologies between fish fins and tetrapod limbs (Owen 1866). The assessment is guided by seven competency questions that aim to capture reasonable user expectations for how an assertion of homology between two anatomical entities should affect subsequent reasoning.

We close with a discussion of implementation of one homology model within the Phenoscape KB, which must balance logical expressivity with the practicalities of scalability across a large data set.

## Materials and Methods

Formalizing the representation of homology requires the use of ontologies and computational reasoning that have been only recently applied to systematics. Thus we provide a glossary to assist readers unfamiliar with these concepts (Box 1).

Box 1. Glossary of terms related to ontologies and computational reasoning

*Annotation*—Statements composed of ontology terms, linked to natural language descriptions such as characters and states.

*Assertion*—A statement in a publication made by an author, typically based on direct specimen observations.

*Class*—A term defined in an ontology representing a concept. There can frequently exist many instances of the concept in the world. In OWL, a class can be thought of as the set of all instances of that concept.

*Entailment*—A logical consequence of reasoning across an ontology. More specifically, the entailments of a formal statement in an ontology are all facts whose truth is necessarily implied by the truth of the statement.

*Ontology*—Ontologies formally represent domain knowledge (e.g., anatomy and taxonomy) in a format that can be understood by a computer, with terms linked by well-defined relationships.

*OWL*—Web Ontology Language, a language standardized by the World Wide Web Consortium (WC3) for defining DL ontologies.

*Property*—A term defined in an ontology that can be used to relate two instances of classes in that ontology. Examples would be “part_of” or “develops_from*.*”

*Property chain*—In OWL, property chains can be used as subproperties, such that two individuals, connected via a chain of relations through some other individual, can be inferred to be directly related via the superproperty. Property chains can be denoted using the “ring” operator, for example, *has_part*$\circ $*part_of* SubPropertyOf *overlaps*.

*Reasoning/semantic reasoning*—The use of logic to derive facts or conclusions that are not explicitly stated in an ontology or model. A reasoner is software that uses formal logic to reach a conclusion.

*Semantic*—Referring to formal meaning of terms.

*Subclass*—also “Subtype.” A term in an ontology that narrows down another (parent) term. Subclasses inherit properties from the parent term; for example, “humerus” is a subclass (or subtype) of “bone,” and thus inherits the property of being composed of bone tissue.

*Subproperty*—A relationship defined in an ontology that is a more specific subtype of its parent relationship. Two instances related by a subproperty can be inferred to be also related by the parent property.

### Logical Models of Homology Assertions

To be used by an OWL reasoner, and thus have an effect on reasoner-driven query resolution, each homology assertion must be translated into OWL axioms. For an explanation of the types of axioms that can be stated within OWL ontologies, see Robinson and Bauer (2011). Modeling in OWL frequently involves a tradeoff between expressivity and reasoner performance on large ontologies. In fact, the OWL language provides three “profiles”—subsets of the language that omit the ability to make certain kinds of statements—which are known to be amenable to more scalable reasoning algorithms (OWL 2 Web Ontology Language Profiles 2012). Because each profile omits different capabilities, each is better suited to particular kinds of modeling tasks. The OWL EL profile is frequently used with complex biomedical terminologies, such as large anatomy ontologies. The availability of efficient EL reasoners such as ELK has been crucial to the application of OWL in the development of ontologies like the Gene Ontology and Uberon (Mungall et al. 2014).

Here, we introduce two alternative logical models for homology: Reciprocal Existential Axioms (REA) and Ancestral Value Axioms (AVA; Table 1). REA is designed to fit within the OWL EL profile. AVA, on the other hand, provides semantics that may potentially be a more exact fit to user expectations of homology but requires reasoning capabilities that are not part of the scalable OWL EL profile. The two models are described here in terms of historical homology; we use *homologous_to* as a shorthand for the “in historical homology relationship with” relation defined in the OBO Relations Ontology. Serial homology is represented using a parallel model employing different relations (below). For precision, we describe axioms using the OWL Manchester Syntax, a formal syntax geared toward human readability (https://www.w3.org/TR/owl2-manchester-syntax/).

**Table 1.** Comparison of the REA and the AVA homology model

Property	REA	AVA	Consequence
Homology relation semantics	All-some	All-all	In AVA, structures are homologous to all subtypes of asserted homologs.
Homology reflexivity is entailed	No	Yes	In AVA, structures are always inferred to be homologous to themselves, such that a query for homologous structures returns the search term and its subclasses.
Homology entailed for individuals	No	Yes	REA is not useful for instance-based queries.
Usable with OWL EL reasoners	Yes	No	Reasoning with the REA model is much more scalable.

As its name states, REA models a homology annotation as a reciprocal pair of axioms. Every instance of the first structure is homologous to some instance of the second structure (i.e., each of the two structures is given a subclass relationship to an existential property restriction using the other structure):

$$\begin{array}{l} \text{"pectoral fin" SubClassOf }\left(homologous\_to\text{ some "forelimb"}\right)\\ "\text{forelimb" SubClassOf}\left(homologous\_to\text{ some "pectoral fin"}\right) \end{array}$$

This model has the advantage that it can be rapidly classified and queried using efficient OWL reasoners implementing the OWL EL profile. REA only enables querying via class expressions, though in the Phenoscape KB this is the most common query approach and thus not a limitation. For example, with the REA model, one can query for all subclasses of the class expression *homologous_to* some “forelimb,” and all instances of this expression. However, as a consequence of the REA model, given a particular instance of “forelimb,” one cannot find other individuals inferred to be homologous to it, because the respective axiom asserts only that (all) instances of “forelimb” are homologous to some instance of “pectoral fin,” not to all instances of it. This is sometimes referred to as “all-some” semantics.

The AVA model introduces, for each homology annotation, an instance (i.e., OWL individual, which we generate programmatically) that represents the ancestral structure from which all instances of the two classes of homologous structures are descended:

$$\begin{array}{l} \text{ectoral fin" SubClassOf }historical\_homology\_member\_of\text{ value }\langle \text{pectoral\_fin\_forelimb\_ancestor}\rangle \\ "\text{forelimb" SubClassOf }historical\_homology\_member\_of\text{ value }\langle \text{pectoral\_fin\_forelimb\_ancestor}\rangle \end{array}$$

Two additional property axioms allow inference of the needed *homologous_to* relationship:

$$\begin{array}{l} historical\_homology\_member\_of\text{ InverseOf }has\_historical\_homology\_member\\ historical\_homology\_member\_of\text{ o }has\_historical\_homology\_member\text{ SubPropertyOf }homologous\_to \end{array}$$

The result of these axioms is an “all-all” semantics, as opposed to the all-some semantics of REA. That is, this model entails for any two instances of “pectoral fin” and “forelimb,” that they are related to each other via a *homologous_to* relationship. Properties with all-all semantics are exceedingly rare, at least in most ontologies encoding biological knowledge domains, because most biologically important relationships can be universally asserted only in one direction. For example, the *part_of* relationship common in anatomy ontologies (such as Uberon) holds between two anatomical entities as A SubClassOf (*part_of* some B), as in humerus SubClassOf (*part_of* some forelimb). The all-some semantics entail that a given instance of humerus is part of one specific forelimb; it is not part of every instance of forelimb. The stronger all-all semantics provided by AVA may more closely match the expectations of a user who asserts historical homology between two anatomical entities. However, the logical expressivity needed for reasoning with AVA requires features, such as inverse property axioms, that are outside of the OWL EL profile, which in practice makes this model much less scalable.

With either model, one can assert homology between structures in a way that is taxonomically more restrictive than implied by the way that the corresponding anatomy ontology terms are defined. To account for such restrictions, we substitute the anatomical entities A and B with taxon-based subclass expressions. More formally, if specifically, entity A occurring in taxon X is homologous to entity B occurring in taxon Y, we substitute A with the class expression “A and *in_taxon* some X” (i.e., those instances of A that are in some instance of taxon X) and B with “B and *in_taxon* some Y” (here using the REA model):

$$\begin{array}{l} \left(\text{A and }in\_taxon\text{ some X}\right)\text{SubClassOf}\left(homologous\_to\text{ some }\left[\text{B and in\_taxon some Y}\right]\right)\\ \left(\text{B and }in\_taxon\text{ some Y}\right)\text{SubClassOf}\left(homologous\_to\text{ some}\left[\text{A and in\_taxon some X}\right]\right) \end{array}$$

Here, X and Y are terms from a taxonomy ontology, for example, Vertebrate Taxonomy Ontology (VTO; Midford et al. 2013). The *in_taxon* relation (RO:0002162) is used throughout Uberon to specify “taxonomic constraints” on anatomical concepts. It is used primarily for automated quality control of annotations and for consistency checking when merging independently developed anatomy ontologies into Uberon.

Different types of homology are differentiated by the specific homology relation used in place of *homologous_to* in the above models. To relate two anatomical terms as historically homologous, we used the relation “in historical homology relationship with” (RO:HOM0000007) from the OBO Relations Ontology (RO; http://purl.obolibrary.org/obo/ro.owl), which is defined as: “Homology that is defined by common descent.” Serially homologous structures were related using the relation “in serial homology relationship with” (RO:HOM0000027), which is defined as: “Iterative homology that involves structures arranged along the main body axis.” These relations are derived from the HOM; http://purl.obolibrary.org/obo/hom.owl), which contains 66 classes representing concepts related to organismal similarity, including homology and homoplasy. Classes of homology from this ontology are mirrored as object properties within RO, providing the relationships needed to assert historical or serial homology between anatomical structures. For testing the AVA model, we used locally defined properties for “historical_homology_member_of” and “has_historical_homology_member” (and corresponding relations for serial homology), as these are not currently defined in the Relation Ontology.

### Biological Expectations for Homology Reasoning

To evaluate the consequences of applying a formal homology relationship between anatomical structures, we establish specific user expectations in the form of competency questions (Grüninger and Fox 1995; Malheiros and Freitas 2017) for the results of a description logic (DL) query of our demonstration ontology. Competency questions are a set of questions that an ontology must answer using the knowledge represented by its axioms (Grüninger and Fox 1995; Malheiros and Freitas 2017). A DL query is an OWL expression logically describing a class for which we want to find its subclasses or instances. Our competency question expressions are modeled using the relations composing the EQ phenotypes (anatomical entity [E] and a quality [Q; Mungall et al. 2007, 2010]) in the test data set but the qualities themselves do not play a role in the homology reasoning. Put another way, *inheres_in* some (*homologous_to* some “pectoral fin”) would subsume any phenotype instances referring to homologs of the pectoral fin. We focus on expectations about how homology is inferred across the broader ontology graph in which the anatomical structures are embedded, for example “to what degree is an assertion of homology propagated to other relationships, such as structural, positional, and developmental ones?”

To our knowledge, this is the first attempt to formalize expectations for homology reasoning in a general manner suitable for evaluating a semantic model. These expectations are framed from the standpoint of a hypothetical user, a comparative evolutionary anatomist who is well-versed in the data that pertain to homology of the structures under consideration. The expectations of this persona guide the general way in which the logic of a homology relationship between two structures propagates beyond them to their parts, types, developmental precursors, developmental products, and other homologs. Although some expectations are clear, and would be so to any biologist (e.g., that the parts of homologous structures are not necessarily homologs), others might be debatable. In these cases, we take a conservative approach wherever homology reasoning might lead to incorrect inferences. For example, some might desire homology reasoning to lead to the conclusion that the developmental precursors of homologous structures are themselves homologs. Given the evidence that this is incorrect in some cases (i.e., developmental precursors of homologs are not themselves homologous), extending the homology reasoning to developmental precursors is not permitted. Thus, homology reasoning in our models involves only subsumption (*is_a*) relations. Reasoning to other relationship types (*develops_from*, *part_of*) would need to be executed via property chain reasoning that is not employed in our homology models. Overall, we take the approach to formulate general expectations for inferred results only for relationships for which the propagation of homology should generally be biologically correct or accepted.

The expectations that are generally applicable to any structure for historical and serial homology queries are the following: In the case of historical homology, queries for historical homologs of a structure are expected to return historical homologs, subtypes of historical homologs, historical homologs of the superclass and its subtype(s), and taxonomically restricted results. Further, queries for historical homologs of a structure are not expected to return parts of the historical homolog, serial homologs, developmental products (i.e., structures that later develop from the historical homolog), or developmental precursors of the historical homolog.

In the case of serial homology, queries for serial homologs of a structure are expected to return serial homologs and subtypes of serial homologs, and serial homologs of the superclass and its subtype(s). Further, queries for serial homologs of a structure are not expected to return parts of the serial homolog, historical homologs of the serial homologs, historical homologs of the superclass and subtype(s), developmental precursors of the serial homolog, or developmental products or developmental precursors of the serial homolog.

### Competency Questions

**Figure 1.**
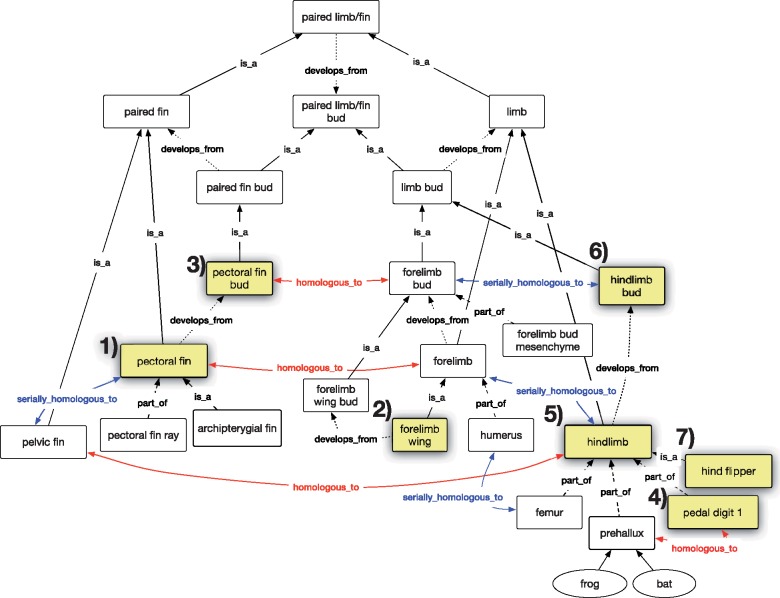
Terms and relationships for structures in the Uberon anatomy ontology pertaining to the paired fins and limbs. Query terms for competency questions 1–7 shown in yellow fill.

**Figure 2.**
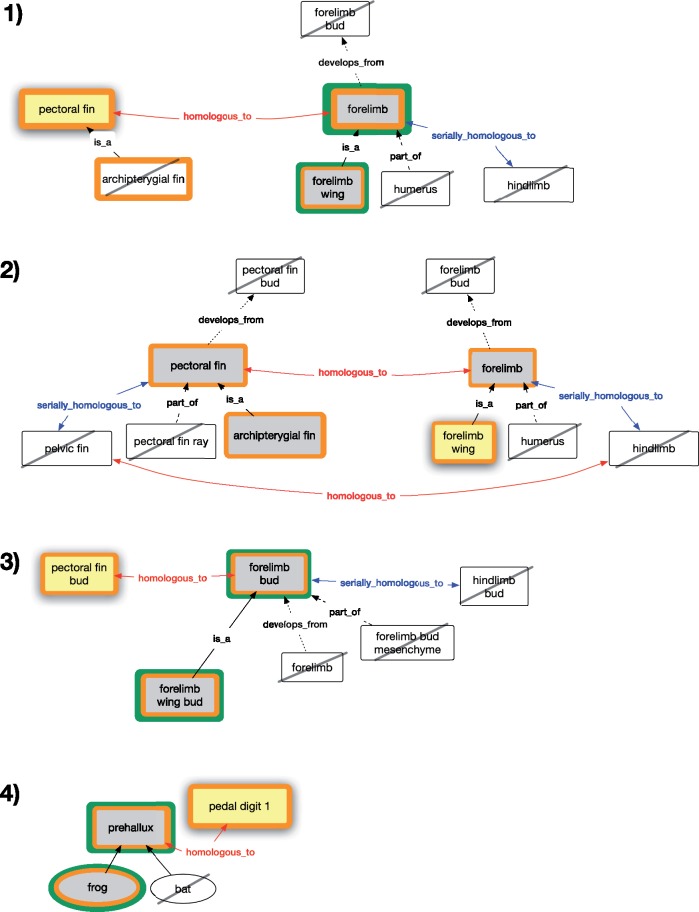
Subgraphs of paired fin and limb terms showing terms pertaining to competency questions 1–4 on historical homology. Query terms (yellow fill), expected classes (grey fill), unexpected classes (black slash), and results from the REA model (green outline) and AVA model (orange outline) are shown for each competency question. In competency question 4, rectangles represent anatomy terms and ovals represent taxonomy terms.

**Figure 3.**
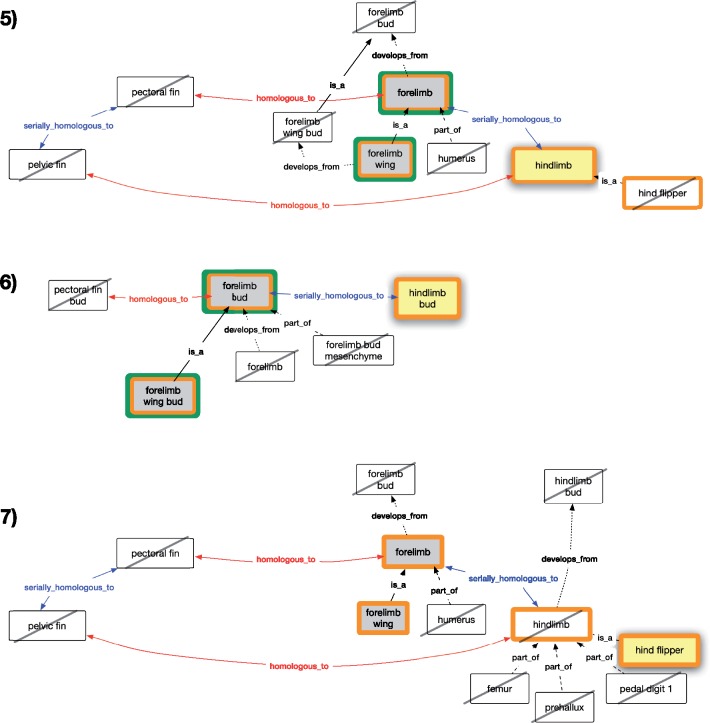
Subgraphs of fin and limb terms showing terms pertaining to competency questions 5–7 on serial homology. Query terms (yellow fill), expected classes (grey fill), unexpected classes (black slash), and results from the REA model (green outline) and AVA model (orange outline) are shown for each competency question.

We created seven competency questions to test the expectations of our biologist persona for the results of queries pertaining to the paired fins and limbs (Fig. 1) using historical or serial homology. The expectations for each query are illustrated in Figures 2 and 3. The OWL expressions for these competency questions are provided in Supplementary material S4 available on Dryad at https://doi.org/10.5061/dryad.0373j7r and also in the homology demonstration file (see Software and Data Availability section).

#### Competency question 1

Our biologist persona expects a phenotype query for historical homologs of “pectoral fin” to return phenotypes for its homolog “forelimb” and its homolog’s subtype “forelimb wing,” as illustrated in Figure 2. They do not expect the query to return phenotypes for parts of the homolog, such as “humerus,” nor phenotypes for serial homologs of the homolog (“hindlimb”). Further, they do not expect it to return phenotypes for the homolog’s developmental precursor “forelimb bud.”

#### Competency question 2

Our persona expects a phenotype query for historical homologs of “forelimb wing” to return phenotypes for “forelimb,” “pectoral fin,” and subclasses of “pectoral fin” such as “archipterygial fin” (Fig. 2). They do not expect the query to return phenotypes for parts (e.g., “humerus” or “pectoral fin ray”) of the homologs, serial homologs (“hindlimb” or “pelvic fin”) of the homologs, and developmental precursors (“forelimb bud” or “pectoral fin bud”) of the homologs.

#### Competency question 3

Our persona expects a query for historical homologs of “pectoral fin bud” to return phenotypes for “forelimb bud” and its subtype “forelimb wing bud” (Fig. 2). They do not expect it to return phenotypes to parts (“forelimb bud mesenchyme”), structures that form later in the course of development (“forelimb”), or serial homologs (“hindlimb bud”) of the historical homologs.

#### Competency question 4

In cases where the homology statement selectively applies to a subset of taxa that possess the anatomical structure, though other taxa may ostensibly possess it as well, our persona expects results for only a restricted set of taxa. They expect a query for the historical homologs of “pedal digit 1” to return “prehallux” phenotypes for only anurans (frogs) and not “prehallux” phenotypes for mammals (bats in Fig. 2). This is because the homology relationship is specific to Anura and pedal digit 1.

#### Competency question 5

From a query on serial homologs of “hindlimb,” our persona expects to find phenotypes for “forelimb” and its subtype “forelimb wing” (Fig. 3). They do not expect the subtype of the search term (“hind flipper”) to be returned, or the historical homologs of the serial homolog (“pectoral fin”) to be returned. Further, they do not expect phenotypes to parts of the homolog’s serial homolog (e.g., “humerus” of the “forelimb”), or their developmental precursor (“forelimb bud” and “forelimb wing bud”).

#### Competency question 6

Our persona expects that a query for serial homologs of “hindlimb bud” would return phenotypes for “forelimb bud” and its subtype “forelimb wing bud” (Fig. 3), but not its developmental product “forelimb,” nor its parts (“forelimb bud mesenchyme”), or its serial homolog (“pectoral fin bud”).

#### Competency question 7

Our persona expects a phenotype query for serial homologs of “hind flipper” to return phenotypes for its serial homolog “forelimb,” and subclasses of “forelimb” such as “forelimb wing,” as illustrated in Figure 3. They do not expect the query to return the superclass of the search term (“hindlimb”), structures for parts (e.g., “humerus,” “femur”) of the serial homologs, historical homologs (e.g., “pectoral fin”) of the serial homologs, or developmental precursors (e.g., “hindlimb bud,” “forelimb bud”) of the serial homologs.

### Annotation of Homology Assertions

Assertions of homology and statements of lack thereof among the skeletal elements of vertebrates were extracted from the phylogenetic literature on teleost fishes and early sarcopterygians (Dececchi et al. 2015), reviews of fin and limb evolution (Hall 2008; Clack 2012), and select papers from the developmental genetic literature (e.g., Shou et al. 2005; Tamura et al. 2011). We systematically sought explicit homology statements between the skeletal elements in actinopterygian fins and sarcopterygian fins and limbs. Though not comprehensive, because the literature in the area of fin/limb evolution is substantial and homology statements specific to many taxonomic groups were not extracted (e.g., between urodele and anuran amphibians), many of the well-known and controversial homologies across fishes and amphibians were captured.

Similar to Cranston et al. (2014), we found that evidence for homology is explicitly asserted in the literature only rarely. This is particularly surprising in the phylogenetic literature, where what is judged to be the same character state represents an explicit hypothesis of putative primary homology among the taxa that share it. Although investigators routinely judge sameness (homology) using criteria of similarity in structure or topographic position, in relation to specific character states, this is rarely explicitly stated. That is, a statement such as “Anatomical feature X in taxa A, B, and C is similar in structure and they are thus considered homologous” is rare in the literature. An additional issue was observed in extracting homology statements from the comparative monographic and fin to limb evolution review literature, in that the focus is often on skeletal elements where homologies are not clear (e.g., radials and digits) as compared with elements such as the humerus or femur, where the homologies are thought to be clear (though rarely explicitly described).

Homology statements were annotated using the appropriate ontologies: anatomy terms using the Uberon anatomy ontology (Haendel et al. 2014) and taxa with the Vertebrate Taxonomy Ontology (Midford et al. 2013). Along with attribution for each statement, we recorded the type of evidence that supported or rejected a historical or serial homology relationship (Dahdul et al. 2010) using the following terms that are types of phenotypic similarity evidence, from the Evidence & Conclusion Ontology (ECO; Chibucos et al. 2014): positional similarity evidence (ECO:0000060), compositional similarity evidence (ECO:0000063), developmental similarity evidence (ECO:0000067), morphological similarity evidence (ECO:0000071), gene expression similarity evidence (ECO:0000075), and structural similarity evidence (ECO: 0000027). Terms for types of phylogenetic evidence (ECO: 0000080) that support homology are available in ECO, though not applicable to the literature referenced in relation to the terms included herein (Supplementary material S1 available on Dryad). We also recorded statements of homology for which a source of the evidence was cited and for which no evidence or source was explicitly given by annotation with the terms “traceable author statement” (ECO:0000033) and “non-traceable author statement” (ECO:0000034), respectively.

Assertions about homology in the literature sometimes also take the form of rejecting or discounting a homology relationship between structures. We recorded these, including the supporting evidence types as per above, using *not_homologous_to*, rather than *homologous_to*, in the relationship column. Although it is possible to encode the negation of a homology relationship in OWL using a Manchester syntax expression such as “not (*homologous_to* some X),” we typically only have these “not” assertions when there is a corresponding is *homologous_to* statement. Adding the “not” annotations as logical axioms would cause reasoning contradictions; we store these annotations as metadata, such that they don’t participate in reasoning.

The fin/limb-specific homology assertions are shown in Table 2. The full collection of homology assertions and associated provenance metadata is publicly available at http://purl.org/phenoscape/demo/phenoscape_homology.owl and in Supplementary material S1 available on Dryad (see Software and Data Availability section). Note that this file is intended to be used in conjunction with referenced ontologies (e.g., Uberon) and includes only the homology axioms, not metadata for individual terms such as label and definition.

**Table 2.** Subset of homology assertions used in the present study pertaining to fins, limbs, and related structures

Entity 1	Taxon 1	Relationship	Entity 2	Taxon 2	Evidence	Attribution
Forelimb	Tetrapoda	*Serially homologous to*	Hindlimb	Tetrapoda	Position	Ruvinsky and Gibson-Brown (2000)
Forelimb bud	Tetrapoda	*Serially homologous_to*	Hindlimb bud	Tetrapoda	Gene expression	Tabin and Laufer (1993) and Hall (2008)
Humerus	Tetrapoda	*Serially homologous_to*	Femur	Tetrapoda	Gene expression	Nelson et al. (1996)
Pectoral fin	Vertebrata	*Homologous_to*	Forelimb	Tetrapoda	NAS	Goodrich (1930)
Pectoral fin	Vertebrata	*Serially homologous_to*	Pelvic fin	Vertebrata	Gene expression	Tabin and Laufer (1993) and Hall (2008)
Pectoral fin bud	Vertebrata	*Homologous_to*	Forelimb bud	Tetrapoda	Developmental similarity	Freitas et al. (2007) and Hall (2008)
Pelvic fin bud	Vertebrata	*Homologous_to*	Hindlimb bud	Tetrapoda	Developmental similarity	Freitas et al. (2007) and Hall (2008)
Pelvic fin	Vertebrata	*Homologous_to*	Hindlimb	Tetrapoda	NAS	Goodrich (1930)
Prehallux	Anura	*Homologous_to*	Pedal digit 1	Tetrapoda	Developmental similarity	Galis et al. (2001)
Prehallux	Anura	*Not homologous_to*	Pedal digit 1	Tetrapoda	Developmental similarity	Fabrezi (2001)
Prehallux	Anura	*Not homologous_to*	Pedal digit 1	Tetrapoda	Developmental similarity	Galis et al. (2001)

*Notes:* Each assertion relates an Entity 1 in Taxon 1 as historically or serially homologous (or not) to an Entity 2 in Taxon 2 based on evidence (annotated with terms from the ECO; Chibucos et al. 2014) cited in the literature (Attribution). In ECO, “NAS” is a type of author statement without traceable support that is used in a manual assertion. The term and identifiers for Uberon terms are: femur, UBERON:0000981 forelimb, UBERON:0002102; forelimb bud, UBERON:0005417; forelimb wing, UBERON:0000024; forelimb wing bud, UBERON:4300230; hindlimb, UBERON:0002103; hindlimb bud, UBERON:0005418; humerus, UBERON:0000976; pectoral fin, UBERON:0000151; pectoral fin bud, UBERON:4300172; pelvic fin, UBERON:0000152; pedal digit 1, UBERON:0003631; prehallux, UBERON:0012136.

We did not include homology axioms from the vHOG (Niknejad et al. 2012), because most of these axioms (as of 23 July 2018) are “self-homologies” with a specific taxonomic scope. For example, vHOG represents the humerus bone as a historically homologous structure within taxon Sarcopterygii. Because in OWL each class is also a subclass of itself, this approach does not yield any additional results (i.e., logical entailments). For the purposes of our investigation, they are redundant with the axioms provided by the anatomy ontology.

### Homology Demonstration File

To evaluate the two homology models and demonstrate how they differ, we assembled a set of phenotypes for fish fins and tetrapod limbs and, as per above, a corresponding set of homology assertions among the relevant entities. Twenty ontology-annotated phenotypes for entities that are types of fin and limb and their literature sources included in the homology demonstration file (see Software and Data Availability section) were drawn from the $>$72,000 gene and taxon phenotypes in the Phenoscape KB. An additional two phenotypes (“forelimb wing bud present,” “forelimb bud mesenchyme present”) were added to the homology demonstration file for testing purposes. This set of 23 fin/limb phenotypes used in the homology demonstration file is shown in Table 3 and in Supplementary material S2 available on Dryad (see Software and Data Availability section). OWL instances representing organism phenotype annotations were created using the Protégé OWL editor, following the Entity–Quality model (Mungall et al. 2007, 2010). For each competency question, we added a named class expression to this OWL file for the purpose of allowing an automated reasoner to infer subsumption of phenotype instances. The homology demonstration file was provisioned with the expected phenotypes as well as phenotypes that would not be expected, because biologists may also have expectations of results that should not be returned (e.g., parts or developmental precursors).

**Table 3.** Subset of fin/limb phenotypes from the Phenoscape KB used in the demonstration file to test the REA vs. AVA homology models

Entity	Quality	Related entity	Species (scientific name)
Archipterygial fin	Present		*Glyptolepis*
Forelimb	Length	Hindlimb	*Eoraptor lunensis*
Forelimb bud	Small		*Mus musculus*
Forelimb bud mesenchyme	Present		*Mus musculus*
Forelimb wing	Structure		*Pteropus giganteus*
Forelimb wing bud	Present		*Gallus gallus domesticus*
Hind flipper	Present		*Callorhinus ursinus*
Hindlimb	Decreased length		*Triadobatrachus massinoti*
Humerus	Decreased length	Trunk vertebra	*Acanthostega gunnari*
Limb	Decreased length		*Dicynodontia*
Manual digit 1	Torsioned		*Xenophrys aceras*
Manus	Has extra parts of type	Phalanx	*Hippopotamus amphibius*
Paired fin bud hypoplastic			*Danio rerio*
Pectoral fin	Position	Cleithrum	*Acestrorhynchus pantaneiro*
Pectoral fin bud aplastic			*Danio rerio*
Pectoral fin ray	Bifurcated		*Colossoma macropomum*
Pedal digit 1	Decreased length	Pedal digit 2	*Dasypus novemcinctus*
Pelvic fin	Located in	Posterior region of body	*Adrianichthys oophorus*
Prehallux	Present		*Myotis lucifugus*
Prehallux	Present		*Callobatrachus sanyanensis*
Small forelimb buds			*Mus musculus*
Small hindlimb buds			*Mus musculus*
Small limb buds			*Mus musculus*

We built a demonstration workflow which produces a file for each model (annotations-ava.ofn and annotations-rea.ofn) that includes homology axioms, phenotype annotations, and relevant axioms from source ontologies. We generated sets of OWL axioms representing homology relationships for each of the two different models (REA and AVA) using Scala scripts included in the phenoscape-owl-tools project (https://github.com/phenoscape/phenoscape-owl-tools). We used the ROBOT command-line tool (http://robot.obolibrary.org) to extract a reduced module of axioms from the Uberon anatomy ontology relevant to the terms used in the demonstration ontology using syntactic locality module extraction (Grau et al. 2007) and to construct a merged ontology file for each homology model. These merged files are small enough to be queried within Protégé using the HermiT OWL-DL reasoner (Glimm et al. 2014), which comes with Protégé and supports the full range of OWL DL expressivity. The demonstration ontology workflow is available on GitHub in the homology-annotations-demo project (https://github.com/phenoscape/homology-annotations-demo).

## Results

### Homology Statements

In total, 46 homology assertions were collected for the paired fins and limbs, including 10 statements pertaining to serial homology. Six positive assertions of homology were contradicted by negative statements of homology. For example, the alular digit in birds was asserted as *homologous_to* manual digit 1 in nonavian tetrapods based on gene expression evidence (Vargas and Fallon 2005; Tamura et al. 2011), whereas these two structures were deemed not homologous based on developmental and morphological similarity (Burke and Feduccia 1997). The most common evidence type recorded was based on development (27 statements), followed by morphological similarity (26 statements), position (20 statements), and gene expression (14 homology statements); 5 statements cited evidence traceable to a different publication, whereas 6 statements did not cite traceable evidence.

### REA Versus AVA Models

Results from REA and AVA models (Figs. 2 and 3) are described in relation to each competency question and in Table 4.

**Table 4.** Results expected by our biologist persona and the results obtained under REA and AVA models for competency questions 1–7

Competency question	Query term	Homology	Expectation	REA results	AVA results
1 (Fig. 2)	Pectoral fin	Historical	Forelimb	Forelimb	Forelimb
			Forelimb wing	Forelimb wing	Forelimb wing
					*Pectoral fin*
					*Archipterygial fin*
2 (Fig. 2)	Forelimb wing	Historical	Forelimb	None	Forelimb
			Pectoral fin		Pectoral fin
			Archipterygial fin		Archipterygial fin
					*Forelimb wing*
3 (Fig. 2)	Pectoral fin bud	Historical	Forelimb bud	Forelimb bud	Forelimb bud
			Forelimb wing bud	Forelimb wing bud	Forelimb wing bud
					*Pectoral fin bud*
4 (Fig. 2)	Pedal digit 1	Historical	Prehallux in anurans (frogs)	Prehallux in anurans (frogs)	Prehallux in anurans (frogs)
					*Pedal digit 1*
5 (Fig. 3)	Hindlimb	Serial	Forelimb	Forelimb	Forelimb
			Forelimb wing	Forelimb wing	Forelimb wing
					*Hindlimb*
					*Hind flipper*
6 (Fig. 3)	Hindlimb bud	Serial	Forelimb bud	Forelimb bud	Forelimb bud
			Forelimb wing bud	Forelimb wing bud	Forelimb wing bud
					*Hindlimb bud*
7 (Fig. 3)	Hind flipper	Serial	Forelimb	None	Forelimb
			Forelimb wing		Forelimb wing
					*Hindlimb*
					*Hind flipper*

*Notes:* Results from AVA that are due to self-homology, subtype, and superclass are denoted in italics. The term names and identifiers from Uberon are: archipterygial fin, UBERON:4200003; forelimb, UBERON:0002102; forelimb bud, UBERON:0005417; forelimb wing, UBERON:0000024; forelimb wing bud, UBERON:4300230; hind flipper, UBERON:4300239; hindlimb, UBERON:0002103; hindlimb bud, UBERON:0005418; pectoral fin, UBERON:0000151; pectoral fin bud, UBERON:4300172; pedal digit 1, UBERON:0003631; prehallux, UBERON:0012136.

#### Competency question 1 results

Both REA and AVA returned the expected phenotypes; AVA additionally returned phenotypes for the search term itself (“pectoral fin”) and its subtype (“archipterygial fin”; Fig. 2 and Table 4). These results are consistent with OWL entailments of the respective models.

#### Competency question 2 results

REA did not return any results from a query for homologs of “forelimb wing.” The same AVA query returned all expected results and additionally phenotypes for the search term itself (“forelimb wing”; Fig. 2 and Table 4). These results are consistent with OWL entailments of the respective models.

#### Competency question 3 results

Both REA and AVA returned “forelimb bud” and “forelimb wing bud”; AVA returned the expected results and additionally “pectoral fin bud” (Fig. 2 and Table 4). These results are consistent with OWL entailments of the respective models.

#### Competency question 4 results

REA returned the expected results; AVA returned the expected results and additionally “pedal digit 1” (Fig. 2 and Table 4). These results are consistent with OWL entailments of the respective models.

#### Competency question 5 results

REA returned the expected results. AVA returned the expected results and additionally “hindlimb” and its subclass “hind flipper” (Fig. 3 and Table 4). These results are consistent with OWL entailments of the respective models.

#### Competency question 6 results

REA returned the expected results; AVA returned the expected results and additionally “hindlimb bud” (Fig. 3 and Table 4). These results are consistent with OWL entailments of the respective models.

#### Competency question 7 results

REA returned no results from a query for serial homologs of “hind flipper.” The same AVA query returned all expected results and additionally phenotypes for the search term itself (“hind flipper”) and its superclass (“hindlimb”; Fig. 3 and Table 4). These results are consistent with OWL entailments of the respective models.

## Discussion

Effectively incorporating homology relationships into anatomy ontologies lays the groundwork for this knowledge to be used in other ontology-based tools and reasoning applications, including candidate gene discovery and phenotypic matrix assembly (e.g., OntoTrace; Dececchi et al. 2015). We developed and evaluated two models for representing historical and serial homology relations using a collection of homology assertions and a set of taxon phenotypes for vertebrate fins and limbs from the KB. The two models that we evaluated reflect an inherent tradeoff between expressivity and computational efficiency. Although there are other ways to represent homology, these two models are sufficient to show biologists the differing logical ramifications. We have surfaced these differences by way of competency questions that force us to specify exactly what a biologist would expect by way of reasoning outcome. These expectations may differ among biologists, and our competency questions are not comprehensive, but we believe that we have provided a foundation that can be built upon by future investigators.

Both of the OWL models, we explored represent a homology assertion as a binary relation. For example, we represented the homology statement as “forelimb wings in birds are *homologous_to* pectoral fins in fishes.” Homology can also be considered as a ternary relation (Bock 1969) which points the two homologs (e.g., forelimb wings, pectoral fins) to a more general reference point—the ancestral structure from which they evolved (in this case “pectoral appendages”) in the named monophyletic group that encompasses them. Bock (1969) argues that this conditional phase describing the condition of the feature in the common ancestor should always be included in any statement about the homology of features. For example, the wings of birds and the wings of bats are homologous as tetrapod forelimbs—or the wings in birds are homologous to the pectoral fins in fishes as vertebrate pectoral appendages. In practice, it is often difficult to conceptualize and describe an ancestral anatomical structure in detail; the only description possible is often only at a high level. For example, the hyomandibula in the jaw of fishes is homologous to the stapes, an inner ear bone in mammals, as a bone of the dorsal hyoid arch in vertebrates. “Bone of the dorsal hyoid arch,” references some bone in a region and is not more informative than an ontological parent class expression such as “endochondral bone that is part of or derived from the hyoid arch skeleton.” That is, in the binary representation, the homologs are also connected to a more general anatomical class, but there it is implied by the structure of the ontology and is thus not necessarily an evolutionary concept.

Further practical difficulties with ternary representation arise in pointing to the common ancestor from which both homologous structures arose. It may not be possible to determine the position of this ancestor if there is incongruence among phylogenetic trees. Even where there is a single robust phylogeny, there may not be a named taxon class or other identifier that corresponds to the last common ancestor. The binary representation does not point to the last common ancestor for the taxa bearing the homologous anatomical structures (Tetrapoda or Vertebrata in the above examples). However, because anatomical annotations are to taxa, the data could potentially be referred to a phylogeny of choice to infer the ancestral taxon. This is currently a challenge because of the lack of a standardized reference system for clades in a tree. An additional reason for representing homology statements using binary relations is because ternary relations are more complicated and awkward to use and query in OWL. However, if desirable and practical in the future, the AVA model could support the ternary representation by specifying an ontological class of the ancestral structure.

### Evaluating Homology Models

Only the AVA model returned all the user-expected results for each competency question (Table 4). In addition, the AVA model also returned the specified query term and any subtypes, because the query term is itself a descendant of the ancestral structure in the model. The AVA model also returns any superclass of a query term for which the homology relationship has been asserted. The subclasses and superclass returned in Competency Questions 5 and 7 were not expected by the user, although entailed by the AVA model. In the REA model, the expected results were not returned for Competency Question 2 (query for historical homologs of “forelimb wing,” Fig. 2) and Competency Question 7 (query for serial homologs of “hind flipper,” Fig. 3). This is a result of our choice to model homology using existential property restrictions of the REA model. For example, the relevant homology axiom for Competency Question 2 states that every “pectoral fin” is *homologous_to* some “forelimb.” It cannot be assumed by an OWL reasoner that the “forelimb” being referred to is a “forelimb wing”; it may be some other subtype of “forelimb.” Thus, under the REA model, no results were returned from this query. In the AVA model, however, the semantics are defined such that “every ‘pectoral fin’ is *homologous_to* every ‘forelimb,”’ and thus “forelimb wing” was returned.

Although the AVA model more closely meets our persona’s expectations for the competency questions, its reliance on more expressive OWL reasoning prohibits its use in practice, e.g., at the scale of a knowledgebase such as the Phenoscape KB. As discussed above in “Logical models of homology assertions,” the REA model is amenable to more efficient reasoning, such as with the ELK reasoner. Furthermore, although some of our persona’s expected results were missing, REA returned no incorrect answers for our test data.

In this study, we have focused on homology models defined in OWL, representing anatomical terms as OWL classes. This is driven in part by requiring a model that works smoothly with ontologies developed as part of the broad biological OBO library, which includes the Uberon anatomy ontology. This choice impacts reasoning requirements in that it favors OWL EL reasoners. It is possible that other forms of modeling would improve scalability and differently impact downstream reasoning but we have not explored these in depth. One possibility might be to represent anatomy as an instance graph, and make use of expressive OWL RL or Datalog rule-based approaches. Such approaches would, however, be a radical departure from our current semantic toolkit.

### Formalizing Homology

Although considerable research and thought has been applied to understanding how homology can be identified and further codified, including suggestions for a semantic framework (Vogt 2017, 2018a,b), general expectations for a semantic model have not been previously formalized. We translated this biological knowledge into the framework of an ontology graph, considering carefully the way in which homology relationships would be expected to propagate along the logical relationships among entities, their subtypes, parts, and developmental precursors and products.

For example, although in some cases the parts of homologous structures might be homologous, they often are not, and thus homology is not propagated through parthood relationships in our models. This is the case even for serial homologs. No biologist has generally surmised, for instance, that skeletal parts of the fish pectoral fins are homologous to those of our forelimbs, though some have suggested homology between specific parts (radials of the fin to humerus of the forelimb). Incorrect inferences are, therefore, not realized in our semantic model. Rather, where applicable, historical, or serial homology must be directly asserted between structures that are parts of homologs. For example, “humerus” (part of the forelimb) and “radial” (part of the pectoral fin) need to be directly asserted as homologs, even if the structures of which they are a part, “pectoral fin” and “forelimb,” are already asserted as homologs.

Another example of limiting homology inference on the basis of biological knowledge comes from developmental biology. Here, we restricted reasoning across development because of the widely recognized disconnect between homology at different levels of biological organization: homology at one level does not necessitate homology at another (Striedter and Northcutt 1991; Roth 1994; Abouheif et al. 1997). There are many examples of homologous structures that develop from nonhomologous developmental precursors (Wagner 1989). For example, Meckel’s cartilage (part of the jaw) in vertebrates is induced differently in amphibians, birds, and mammals (Wagner 1989). *Vice versa*, there are many examples of nonhomologous structures whose development is similar, for example, under the control of orthologous genes. For example *distal-less* regulates outgrowth of the limbs of insects and vertebrates, but phylogenies nearly conclusively reflect the independent evolution of limbs in these taxa (i.e., that they are not historical homologs; Panganiban et al. 1997). Because of this lack of homology correspondence across biological levels, the desired outcome from a query for historical homologs of “pectoral fin bud” would be “forelimb bud” or “forelimb wing bud,” but not the product of further bud development, that is, “forelimb” or “forelimb wing.” *Vice versa*, the desired outcome from a query for historical homologs of “pectoral fin” would be “forelimb” and its subtype “forelimb wing,” but not their developmental precursors “forelimb bud” and “forelimb wing bud.”

We also took a conservative approach to extending reasoning across multiple types of homology relationships. For example, although “hindlimb” is serially homologous to “forelimb,” and “hindlimb” is historically homologous to “pelvic fin,” a query for serial homologs of “hindlimb,” returned its serial homolog “forelimb,” but not the historical homolog of forelimb, i.e., “pectoral fin.” Thus, a serial homology search does not extend to historical homologs of the serial homolog, and likewise an historical homology search does not extend to serial homologs of the historical homolog.

### Homology Assertions Must Be Specific

In initial tests of the reasoning based on homology assertions from the literature, we discovered that homology axioms involving general, i.e., less specific, grouping terms can return unexpected results. For example, although it is accepted that the paired fins of fishes are homologous to the limbs of terrestrial vertebrates, when this statement is translated into a homology assertion (“paired fin” *homologous_to* “limb”), the queries involving the more specific subtypes of these terms yield some results that are more general than expected. Under the AVA model, a query for homologs of “pectoral fin” return both “forelimb” and “hindlimb” because of the semantics of the homology axiom: *every* “paired fin” *homologous_toevery* “limb.” Here, because pectoral fin is a type of paired fin, and under the relationship where paired fin is homologous to limb, the outcome includes both subtypes of “limb,” the forelimb (true historical homolog) and hindlimb (not historical homolog). In contrast, the REA model only returned “forelimb” (and subtype “forelimb wing”), because the semantics for this model (every “paired fin” *homologous_to* some “limb”) asserts that only some instances of “limb” are homologous to “paired fin.” Because pectoral fin also has a homology assertion to forelimb, only forelimb is returned.

Although in many cases, it may suffice for a user to query for serial homologs by using a shared parent term (e.g., a query for “vertebra” returns “vertebra 1,” “vertebra 2,” “vertebra 3,” etc. …), in other cases, explicit homology axioms are needed to relate serial homologs. For example, “humerus” and “femur” need an explicit homology axiom because these terms do not share a common parent term in Uberon. Other types of iterative homology (Roth 1984), that is, between bilaterally (e.g., vertebrates) or radially symmetric (e.g., echinoderms) structures or male vs. female organisms, also require explicit serial homology axioms. For example, although terms for structures between right and left sides of the body are subtypes of the more general structure (e.g., “right preopercle” and “left preopercle” are subtypes of “preopercle”), homology between them needs to be asserted. Without such specification, searches for these types of iterative homologs fail (e.g., a search for homolog of the “right preopercle” does not return the expected result, i.e., “left preopercle”).

### Homology Grouping Classes

The Uberon anatomy ontology contains 10 explicit “grouping classes” primarily driven by homology (as opposed to structure, function or position; Haendel et al. 2014). These are high level classes of “nearly certain” homology that were historically developed for Uberon to ensure that users received expected results from data queries without having to explicitly include homology assertions and a model that implements them. For example, a user query to “paired limb/fin” would return “paired fin” and “limb” (Fig. 1). These grouping classes are designated with the “in_subset: homology_grouping” tag, but are not logically related as homologous and do not include evidence or attribution. Nine of these 10 classes are relevant to the fin/limb collection of homology assertions assembled here: “paired limb/fin bud” UBERON:000435; “limb/fin segment” UBERON:0010538; “paired limb/fin cartilage” UBERON:0007389; “paired limb/fin skeleton” UBERON:0011582; “pelvic appendage” UBERON:000470; “paired limb/fin” UBERON:0004708; “pectoral appendage” UBERON:0004710; “paired limb/fin field” UBERON:0005732, and “bone of free limb or fin” UBERON:0004375. These grouping classes do not affect the outcome of the reasoning (see Supplementary materials S3 available on Dryad; see Software and Data Availability section).

### Disabling Anatomical Homology Relations to Discover Deep Homology

The discovery of similar anatomical features that arose independently in evolution and yet are underlain by homologous genes and networks has given pause to many investigators focused on homology at the structural level. Such highly conserved genetic regulation, termed “deep homology” (Shubin et al. 1997, 2009) reflects not only the deep continuity of fundamental circuitry across long stretches of evolution, but also its co-option to generate similar anatomical structures that are nonhomologous. The extent of deep homology across life is unclear, and it will be necessary to make many comparisons of similar structures across diverse organisms to gauge if it is the rule or the exception. Such a research program would be enhanced by the ability to conduct taxonomically broad similarity searches in a knowledgebase such as the one used here. The results of interest, in this case, would be structures that do not owe their similarity to historical or serial homology, such as fly wings, vertebrate limbs, and beetle horns as “appendages,” or the light-sensing organs of arthropods, mollusks, and vertebrates as “eyes.” Thus, implementation of the homology axioms described herein may be useful in providing either a negative or positive filter for search results, depending on the application.

### Implementation in the Phenoscape KB

We have incorporated historical and serial homology reasoning in the Phenoscape KB, where it allows discovery of structures that are related because of common ancestry. Fully implementing homology queries, however, still remains a challenge owing to the limitations of OWL reasoning. In the Phenoscape KB, for example, the more computationally feasible REA model of homology was implemented. However, given the size of the anatomy and phenotype ontologies used by Phenoscape, even with REA, OWL reasoning on the complete terminology is only feasible using fast EL reasoners such as ELK (Kazakov et al. 2014). Although we ultimately select and deploy a model that satisfies basic reasoning, we expect that it can and will be optimized for different purposes and as computational methods evolve to represent uncertainty, hierarchical trait dependencies, and other variables (Tarasov 2019).

In the Phenoscape KB, user queries are currently restricted to positive homology assertions for both historical and serial homologs because contradictory statements cannot be used in reasoning. We previously envisioned enabling a user to choose the specific set of homology assertions to inform their searches (Dahdul et al. 2010). Translating this into a functional model, however, is challenging because of the difficulty of representing conflicting statements (i.e., both *homologous_to* and not_*homologous_to* for the same pair of anatomical entities) within ontologies. Part of this challenge is that reasoning required to handle *not_homologous_to* annotations is not implemented in the Phenoscape homology model. This is because these negative homology assertions are nearly always paired with disagreeing positive homology assertions. Including contradictory assertions in the logical definition of a single anatomical class would render that class unsatisfiable and thus unusable for data retrieval. However, *not_homologous_to* relations are displayed in the metadata in the KB for anatomical terms. Although logically representing both positive and negative assertions might potentially serve the avid and discriminating comparative anatomist, enabling such choices would not necessarily be relevant to users from many other backgrounds.

### Modifying Homology Assumptions On-the-Fly

Consensus concerning the homology of many structures may never be achieved, as different lines of evidence can point in opposing directions. As described above, whether the first “finger” of birds is homologous or not to that in dinosaurs is a well-known example of conflicting evidence. Although we relate homology assertions herein to the data that support them by annotation with homology evidence codes (Dahdul et al. 2010) from the ECO (Chibucos et al. 2014; Giglio et al. 2019), they are not implemented in Phenoscape for customized homology reasoning. It may be desirable in the future, however, to allow user selection of homology assertions based on these codes. For instance, only homology relationships that are backed up by particular lines of evidence (e.g., “similarity of development”) might be chosen, or perhaps only homology that is supported by all the evidence (no conflict). Enabling individualized selection of homology relationships would alter the reasoning and thus the derivative products of knowledgebases. One would expect different sets of phenotypes to be reasoned, e.g., using OntoTrace (Dececchi et al. 2015), and aggregated based on how similarity is treated, i.e., whether judged homologous or not. In turn, products derived from these phenotypes will be affected. These include hypotheses of evolution (phylogenies), candidates for genetic control elements (Hiller et al. 2012) and genes (Edmunds et al. 2016), or even phenotype-based genomic identification (Lippert et al. 2017) for biodiverse species. Because of the iterative nature of homology hypothesis development, these products may provide new evidence for the common ancestry versus convergent nature of particular features. In summary, enabling machines to reason the various types of similarity (evolutionary, structural, functional, etc.) is a challenging but promising area for future work in the area of phenotype-driven knowledge discovery.

## Data Availability

A snapshot of the code available at https://github.com/phenoscape/phenoscape-owl-tools and used in this article is available from Zenodo: https://doi.org/10.5281/zenodo.2605467. The homology demonstration file is available at https://github.com/phenoscape/homology-annotations-demo and the version used in this article is archived at Zenodo (https://doi.org/10.5281/zenodo.3454786). These files can be run in Protégé with the HermiT reasoner (Glimm et al. 2014). Supplementary material S1–S4 available on Dryad.

## Supplementary Material

Data available from the Dryad Digital Repository: https://doi.org/10.5061/dryad.0373j7r
